# Calcium Phosphate Delivery Systems for Regeneration and Biomineralization of Mineralized Tissues of the Craniofacial Complex

**DOI:** 10.1021/acs.molpharmaceut.2c00652

**Published:** 2023-01-18

**Authors:** Darnell L. Cuylear, Nafisa A. Elghazali, Sunil D. Kapila, Tejal A. Desai

**Affiliations:** † Graduate Program in Oral and Craniofacial Sciences, School of Dentistry, University of California, San Francisco, California 94143-2520, United States; ‡ Department of Bioengineering and Therapeutic Sciences, University of California, San Francisco, California 94143-2520, United States; § UC Berkeley - UCSF Graduate Program in Bioengineering, San Francisco, California 94143, United States; ∥ Section of Orthodontics, School of Dentistry, University of California, Los Angeles, California 90095-1668, United States; ⊥ Department of Bioengineering, University of California, Berkeley, California 94143-2520, United States; # School of Engineering, Brown University, Providence, Rhode Island 02912, United States

**Keywords:** calcium phosphates, drug delivery, craniofacial bone defects, dentin, dental decay, intrafibrillar mineralization, biomineralization

## Abstract

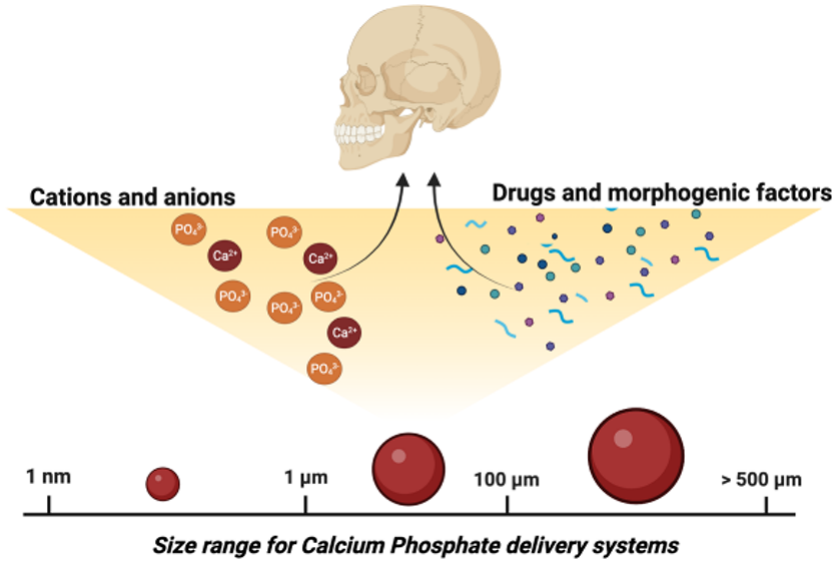

Calcium phosphate (CaP)-based materials have been extensively used for mineralized tissues in the craniofacial complex. Owing to their excellent biocompatibility, biodegradability, and inherent osteoconductive nature, their use as delivery systems for drugs and bioactive factors has several advantages. Of the three mineralized tissues in the craniofacial complex (bone, dentin, and enamel), only bone and dentin have some regenerative properties that can diminish due to disease and severe injuries. Therefore, targeting these regenerative tissues with CaP delivery systems carrying relevant drugs, morphogenic factors, and ions is imperative to improve tissue health in the mineralized tissue engineering field. In this review, the use of CaP-based microparticles, nanoparticles, and polymer-induced liquid precursor (PILPs) amorphous CaP nanodroplets for delivery to craniofacial bone and dentin are discussed. The use of these various form factors to obtain either a high local concentration of cargo at the macroscale and/or to deliver cargos precisely to nanoscale structures is also described. Finally, perspectives on the field using these CaP materials and next steps for the future delivery to the craniofacial complex are presented.

## 1 Introduction

The craniofacial complex contains three subsets of mineralized tissues (bone, dentin, and enamel) that have complex morphologies and distinct functions critical to human health. Of these tissues, bone and dentin have some regenerative properties. However, diseases and injuries affecting these mineralized tissues such as fractures, osteomyelitis, osteonecrosis, tooth decay, and/or periodontitis can lead to severe consequences to the integrity of the mineralized tissue often requiring surgical intervention.^1−5^ The limitations of current surgical approaches have led the field of regenerative medicine and tissue engineering to create new methods to regenerate bone and some components of teeth (mainly dentin) to restore their functionality and structural integrity.

Within the realm of mineralized tissue engineering, the field has focused on approaches that consider the following five factors: Biomaterial approaches should 1) mimic the native components of the extracellular matrix niche; 2) facilitate the differentiation of cells toward a desirable phenotype; 3) recruit osteogenic or odontoblast cells to lay down the organic/inorganic matrices and, with bone, osteoclastic cells to remodel that matrix; 4) provide sufficient vascularization to meet the growing tissue nutrient supply and clearance needs; and 5) facilitate biomimicry by recapitulating known biological processes to restore the tissue to its native structure.^6,7^ Considering these factors, a desirable approach would be a biocompatible material that can deliver relevant cargos like morphogens or ions to provide a favorable local microenvironment for the regeneration and/or biomineralization of craniofacial mineralized tissues. A biomaterial that satisfies a majority of these factors is calcium phosphate (CaP) drug delivery systems. CaP bioceramics are the most studied bone substitutes due to their compositional similarity to native bone, excellent biocompatibility, inherent osteoconductivity, and at times, osteoinductivity.^8^ Although CaPs have been extensively studied in the form of cements, CaP particles (CaPPs) such as microparticles, nanoparticles, and amorphous calcium phosphate (ACP) nanodroplets are an exciting approach for size-dependent mineralized tissue regeneration.

**Figure 1:**
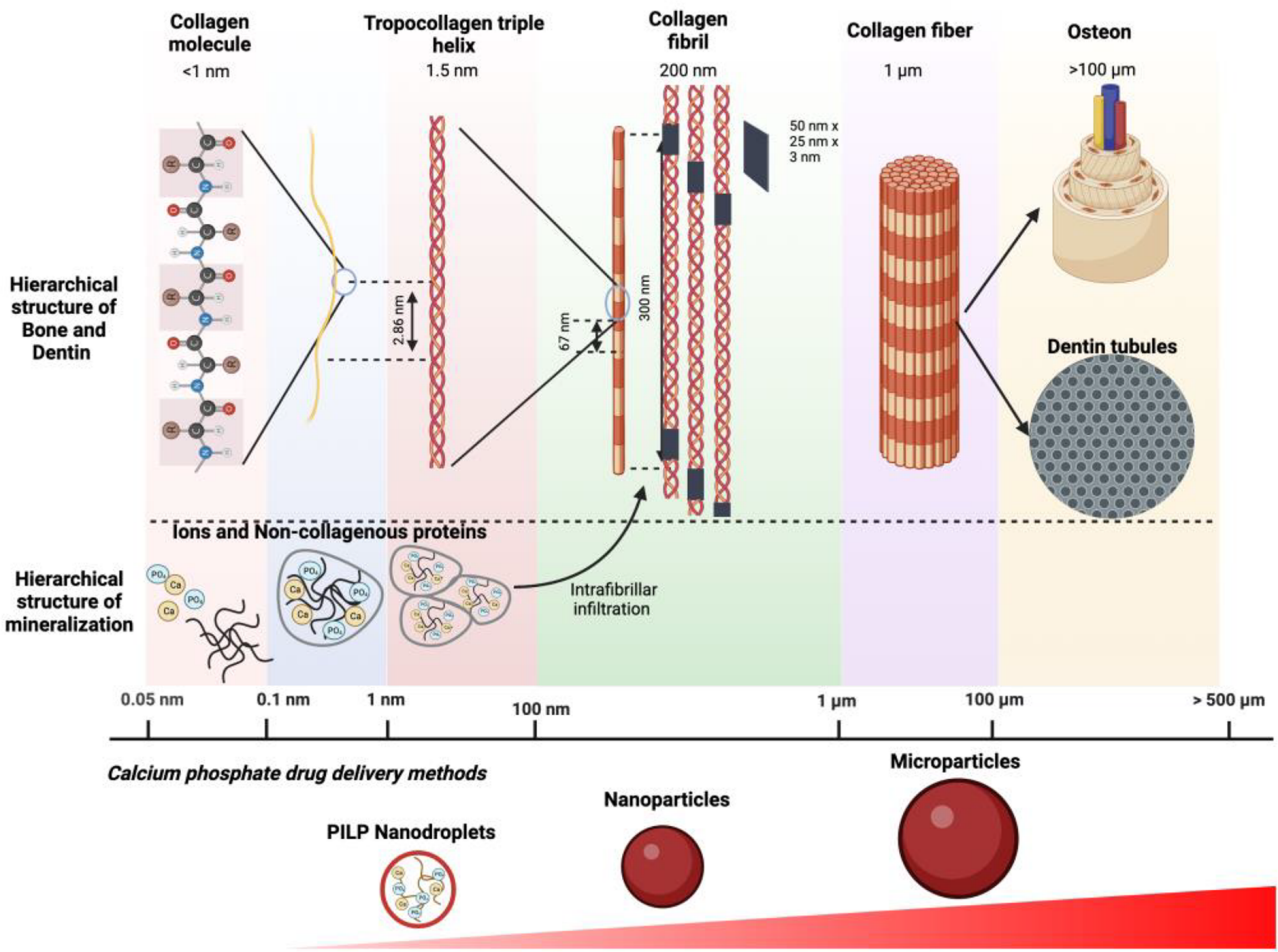
Hierarchical structure and organization of Type-I collagen that composes bone and dentin tissues. Below, various sized drug delivery systems for bone and dentin applications.

Craniofacial bones and dentin are hierarchical tissues with a unique composition of an organic type-I collagen matrix interspersed with inorganic carbonated CaP nanostructures. These CaP nanostructures reinforce the collagen fibrils, giving rise to stiff materials that can withstand loads. Thus, from a biomimetic approach, being able to target these various size scales to deliver either 1) drugs to control cellular recruitment and activity at the microstructure or 2) precursors, such as calcium and phosphate ions, to infiltrate the nanostructures to facilitate biomineralization would be ideal for the complex tissues within the craniofacial complex (Figure 1). The goal of this review is to describe the use of CaPPs with different phase compositions and structures for drug delivery to mineralized tissues in the craniofacial complex, focusing on research published in the last 10 years. First, we address the use of CaPPs microparticles and nanoparticles in their various CaP phase compositions to tune drug delivery and their specific advantages for craniofacial bone regeneration and biomineralization. We then evaluate a special class of amorphous calcium phosphates (ACP) that are stabilized through a process known as polymer-induced liquid precursors (PILPs) that can mimic noncollagenous proteins and infiltrate type-I collagen fibrils. Second, we address the same size form factors, CaP phase compositions, and ACP-PILP delivery for dentin repair and biomineralization.

## 2 Calcium Phosphate (CaP) Delivery Systems for Craniofacial Bone Regeneration and Biomineralization

To treat bone defects within the craniofacial complex, surgeons typically utilize sintered or self-setting cement made of CaP to fill defect sites.^9^ CaP is an excellent material used for bone regeneration due to its similarity to the inorganic bone matrix, inherent biocompatibility, and osteoconductive surface. In addition, CaPs have extensive utility as delivery systems for bone tissue engineering applications, especially in the form of cements, which have been extensively reviewed.^10,11^ Although CaP cements are the most used material, due to the rigidity of the material, surgeons must remove healthy bone to expose areas large enough for the implant. This can contribute to increased bone loss, trauma to surrounding healthy tissue, and a higher risk of contamination at the defect site–further exasperating the invasiveness and risk to the patient.^12^ By using smaller microparticles, nanoparticles, or liquid-like amorphous nanodroplets of CaP, the targeting for defect repairs in the craniofacial complex may be improved. A key advantage of CaP particles (CaPPs) is their tunability in their phase composition, size, release rates, and dissolution rates (to match regeneration rates), as well as utility for local and targeted drug delivery upon implantation and injection, especially in small craniofacial defect sites. Therefore, while challenging, the ability to better target smaller craniofacial defects using minimally invasive and innovative drug delivery approaches can lead to improved healing outcomes and greater aesthetic advantages.^13^ Here, we will review the recent work and advances in the use of CaPPs at the micro- and nanoscale for tuning drug delivery and craniofacial bone repair.

### 2.1 CaP Microparticles for Craniofacial Bone Regeneration

CaPPs in the form of microparticles have advantages over nanoparticles for drug delivery because their larger size facilitates localization postimplantation, which lowers the likelihood of crossing biological barriers while still maintaining high local drug concentrations.^14,15^ CaPPs are used in a variety of sizes as well as phases. The most common CaP phase is hydroxyapatite (HAP). HAP is the most stable CaP phase under physiological conditions and has slow dissolution kinetics. The second most common phase is tricalcium phosphate (TCP), which has a higher dissolution rate but has been thought to dissipate too quickly for bone growth. Other phases include dicalcium phosphate dihydrate (DCPD), amorphous calcium phosphate (ACP), monocalcium phosphate monohydrate (MCPM), tetracalcium phosphate (TTCP), and octacalcium phosphate (OCP), all of which are underutilized for drug delivery in the craniofacial complex (Table 1). Given the strong correlation between dissolution and drug release, phase composition plays a critical role in drug release and downstream biological effects.^10,11,16^ Depending on application, biphasic systems can be utilized to overcome the inherent limitations of a monophasic system. Here, we examine the advances in both monophasic and biphasic systems to control drug delivery and their applications for craniofacial bone repair (Table 2).

**Table 1:** Main CaP Phases^a^

Phase	Chemical Formula	Space Group	*p*K_sp_ at 37 °C	Ca/P Molar Ratio
MCPA	Ca(H_2_PO_4_)_2_	Triclinic P^l̅^	1.14	0.5
MCPM	Ca(H_2_PO_4_)_2_H_2_O	Triclinic P^l̅^	1.14	0.5
DCPD	CaHPO_4_2H_2_O	Monoclinic l_a_	6.6	1
DCPA	CaHPO_4_	Triclinic P^l̅^	7.0	1
β-CPP	Ca_2_P2O7	Tetragonal P4_1_	18.5	1
ACP	Ca_3_(PO_4_)_2_nH_2_O	/	25	1.3–1.5
α-TCP	Ca_3_(PO_4_)_2_	Monoclinic P2_1_/a	25.5	1.5
β-TCP	Ca_3_(PO_4_)_2_	Rhombohedral R3cH	29.5	1.5
TTCP	Ca_4_(PO_4_)_2_O	Monoclinic P2_1_	37.5	2
OCP	Ca_8_H_2_(PO_4_)_6_ 5H_2_O	Triclinic P^l̅^	97.4	1.33
HAP	Ca_10_(PO_4_)_6_(OH)_2_	Pseudo-Hexagonal *P6*_*3*_*/m*	117.3	1.67

a MCPA, monocalcium phosphate anhydrous; MCPM, monocalcium phosphate monohydrate; DCPD, dicalcium phosphate dihydrate; DCPA, dicalcium phosphate anhydrous; CPP, calcium pyrophosphate; ACP, amorphous calcium phosphate (data pertain to the phase obtainable at pH 9–11); TCP, tricalcium phosphate; TTCP, tetracalcium phosphate; OCP, octacalcium phosphate; HAP, hydroxyapatite. Reproduced with permission from ref (16). Copyright 2022 John Wiley and Sons.

**Table 2:** Summary of Calcium Phosphate Micro- and Nanoparticles for Bone Regeneration and Biomineralization

phase	size	defect model	preparation method	method of release modulation
HAP	>500 μm	calvarial^30,52^	wet precipitation^30,52^	none
	500–100 μm	calvarial^20,31,39^	glass conversion^20,31,33,39^	ion substitution^20^
				polymer coating^31^
				heat treatment^33^
	100–1 μm	cavarial^25^	water-in-oil emulsification^22^	ion substitution^22^
		Achilles tendon^18^		
		femoral condyle^26^	hydrothermal^23,24,26,27,32,36−38^	ion substitution^23,24,26,27^
				polymer coatings^36−38^
			wet precipitation^28^	ion deficiency^28^
			commercial^18,19,29^	mineral coatings^18,19,29^
	>200 nm	none	flame spray pyrolysis^62^	surface area^62^
			hydrothermal^79^	dissolution^79^
			wet precipitation^71^	polymer coating^71^
	200–1 nm	type 1 diabetes^72^	water-in-oil emulsification^74^	lipid coating^74^
		tumor^74^	hydrothermal^75,80,81^	lipid coating^75^
				ion substitution^80^
				surface structure^81^
			wet precipitation^60,67,68,70,72^	dissolution^60^
				surface structure^67,68,70^
				polymer coating^72^
TCPs	>500 μm	calvarial^21,41,42,52^	wet precipitation^52^	none
			commercial^21,41,42^	mineral coatings^21^
	500–100 μm	calvarial^43^	water-in-oil emuslisification^43^	particle size ratio^43^
	100–1 μm	none	solid-state reaction^50^	surface area^50^
	>200 nm	none	hydrothermal^79^	dissolution^79^
OCP	>500 μm	calvarial^52^	water-in-oil emulsification^49^	none
			wet precipitation^52^	
	500–100 μm	calvarial^53^	wet precipitation^53^	none
	100–1 μm	none	hydrothermal^48^	surface area^48^
			wet precipitation^47,50,51^	hydrogen bonding^47^
				surface area^48,50^
				crystallinity^51^
	200–1 nm	none	wet precipitation^68^	surface structure^68^
BCP	500–100 μm	patellar defect^58^	water-in-oil emulsification^58^	collagen infiltration^58^
	100–1 μm	none	ultrasonic spray pyrolysis^59^	electrostatic interactions^59^
			wet precipitation^57^	surface area^57^
	>200 nm	mandible^82^	liquid-phase precipitation^82^	none
	200–1 nm	calvarial^76^	hydrothermal^76^	surface structure^76^
		femur^83^	wet precipitation^83^	none
ACP	>200 nm	none	ultrasound irradiation^77^	electrostatic interactions^77^
	200–1 nm	tumor^73^	water-in-oil emulsification^73^	lipid coating^73^
			methanolic conditions^78^	dissolution^78^
			wet precipitation^60^	dissolution^60^
DCPA	>200 nm	none	hydrothermal^64,79^	surface structure^64^
				dissolution^79^
	200–1 nm		wet precipitation^68^	surface structure^68^

#### 2.1.1 Hydroxyapatite (HAP) Microparticles

**Figure 2:**
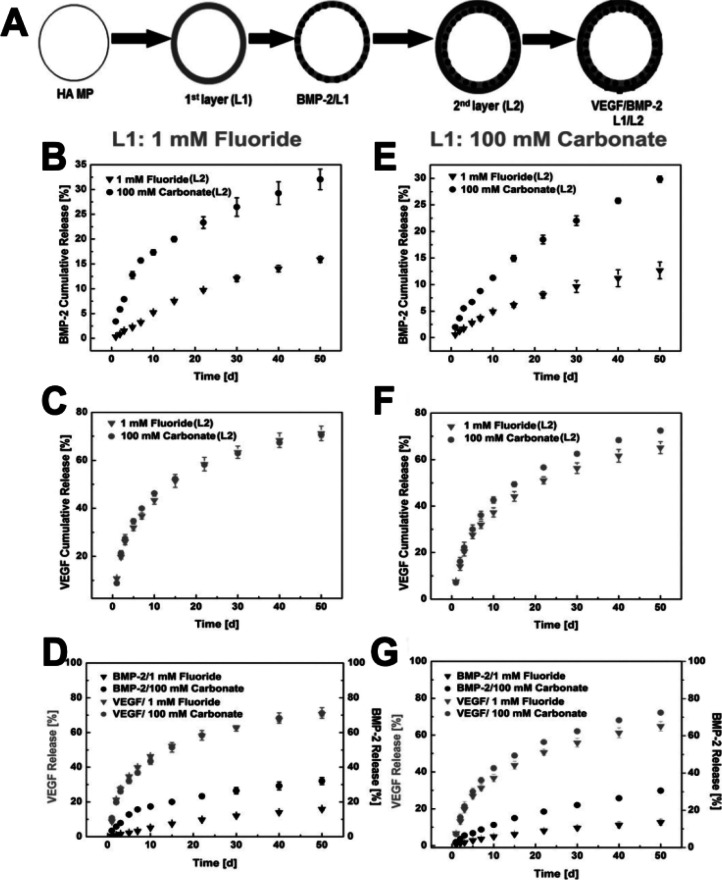
Tunable dual growth factor delivery from hydroxyapatite microparticles. (A) Schematic of hydroxyapatite microparticles (HA MP) mineral coating with layer 1 (L1), then loaded with BMP-2 (BMP-2/L1). The second layer (L2) of mineral was coated on L1 by incubating HA MPs in mSBF, then VEGF was bound on the second layer of the mineral coating. (B–D) 1 mM fluoride mineral coating as L1 and % release: (B) BMP-2 release from L1, (C) VEGF release from L2, and (D) BMP-2/VEGF dual release. (E–G) 100 mM carbonate mineral coating as L1: (E) BMP-2 release from L1, (F) VEGF release from L2, and (G) BMP-2/VEGF dual release. Each condition was tested with 1 mM fluoride or 100 mM carbonate as the 2nd layer. Reprinted from ref (19). Copyright 2022 John Wiley and Sons.

HAP, the most widely used CaP, has been employed to tune the delivery of various drugs and growth factors. HAP has an exceptional ability to induce precipitation of carbonated apatite on its surface due to its bioactivity,^17^ which can be leveraged to tune the release of cargo from HAP microparticles. By using modified simulated body buffer (mSBF) solutions with high concentrations of carbonate, ionic species in the solution can precipitate to the surface of the particles creating a layer of carbonated apatite that exhibits nanoscale features.^18−20^ The ability of carbonated apatite coatings to tune drug delivery is driven by the destabilization of the HAP crystal lattice, resulting in increased dissolution of the particle.^21^ Interestingly, using a layer-by-layer approach, Yu et al. demonstrated that adding carbonated apatite coatings with different dissolution profiles could temporally control the release of both single and dual growth factors over time (Figure 2).^19^ In addition, doping other ion species (i.e., fluoride and magnesium) into the mineral layers decreased the dissolution of the particle, effectively slowing the release of different growth factors in each apatite layer for as long as 40 days. Other ion doping and substitution methods to improve loading efficiency and alter drug release have been implemented with iron,^22^ strontium,^23,24^ magnesium,^25^ zinc,^26^ and silicon,^27^ as well as calcium deficiencies.^28^ In addition to providing controlled release, carbonate coatings can protect and stabilize protein activity in harsh environments on HAP microparticles and other clinically used materials like sutures, further expanding the utility of HAP and carbonated apatite coatings as drug delivery systems.^18,29^

Although HAP has numerous methods to tune drug delivery and modulate cargo release behavior, HAP exhibits poor resorption after implantation *in vivo.* To address this limitation, carbonation of the HAP crystal lattice has shown to also be beneficial in facilitating resorption and dissolution of HAP particles postimplantation.^20,30^ Xiao et al. used bioactive borate glass as a template that could subsequently be converted to HAP with varying ratios of carbonate substitutions.^20^ Using unmodified and carbonated HAP particles in a calvaria defect, carbonated HAPs significantly improved bone formation with fewer particles in the defect site, indicating improved dissolution and susceptibility to resorption. Interestingly, when bone morphogenetic protein-2 (BMP-2) was loaded and released from both particles, there was a further decrease in the number of particles in the defect site. These findings were attributed to the dual effects BMP-2 has on signaling pathways involving both osteoblast and osteoclast formation. These findings have implications on understanding the delivery cargo and how it can, directly and indirectly, affect CaP delivery systems’ retainment and degradation.

Another emerging class of HAP-phase drug delivery methods are hollow hydroxyapatite microparticles.^20,31−34^ Hollow HAP are microparticles with a hollow lumen and mesoporous shell, where the former facilitates drug loading and the latter controls drug release. Currently, hollow HAP are mainly produced by 1) conversion of bioactive glasses to HAP via incubation in a phosphate-rich source and 2) hydrothermal conversion of calcium carbonate into HAP at high temperatures. Additional high heat treatments after hollow HAP conversion allow the outer shell to sinter, leading to decreased pore size and slowed, sustained release of cargos.^33^ In other cases, due to the inherent electrostatic charge on HAP, synthetic polymers can be coated on to the surface to provide alternative modes of slowing release from hollow HAP through layer-by-layer fabrication.^35^ Common polymers include polystyrene sulfonate,^36^ alginate,^37^ poly(lactic-co-glycolic) acid (PLGA),^31^ chitosan,^37,38^ and hyaluronic acid.^38^ For craniofacial bone regeneration, hollow HAP microparticles are an effective method for delivering BMP-2^31^ and transforming growth factor beta (TGF-β)^39^ for improved bone regeneration. Using bioactive glass converted to hollow HAP microparticles, Xiao et al. obtained PLGA coatings with different thicknesses that controlled the release of BMP-2.^31^ With increasing concentrations of PLGA from 5 mg/mL to 200 mg/mL, the total release of BMP-2 was between 1.5 and 1% relative to the uncoated hollow HAP, which exhibited 1.8%. When implanted in calvarial defects, BMP-2-loaded hollow microparticles with 50 mg/mL PLGA coatings significantly improved bone healing, presumably due to the increased osteoconductive surface exposure and adequate release kinetics. Notably, work comparing individual hollow HAP particles to 3D scaffolds showed improved bone regeneration in calvarial defects with individual HAP particles, suggesting that CaPPs may be more suitable for craniofacial bone repair.^39^ However, a limitation of bioactive glass conversion is the size of the particle. In most studies, HAP particles are greater than 100 μm in diameter, which are ideal for large defects but may not be as effective in smaller sites like alveolar bone due to tissue size constraints. To overcome this, investigation into the synthesis of smaller hollow HAP particles would be useful to improve delivery to sites that are limited by volume. Additional work in this area could lead to rational delivery methods to improve bone healing within tissue-constrained sites.^34^

#### 2.1.2 Other CaP Phase Microparticles

A major focus in the field of CaPP-driven bone regeneration is optimizing the release of drug and bioactive molecules. This has led to an approach of tuning CaPP phase composition to improve the degradability and dissolution of the particles and subsequent release of cargo.

To optimize degradation and dissolution, various studies have leveraged tricalcium phosphates (TCPs). TCPs are divided into alpha (α)- and beta (β)-phases, with the difference owing to the crystal structure (i.e., monoclinic vs rhombohedral space groups). In addition, α-TCP is less stable and has a faster dissolution rate compared to β-TCP.^40^ Using α-TCP microparticles and simvastatin as the cargo, Nyan et al. obtained high loading efficiencies (>93%) of the drug with initial burst release (∼25%) and extended-release of up to 14 days.^41^ In calvarial defects, simvastatin-loaded α-TCP exhibited a pronounced effect on bone regeneration in the defect site that was partially attributed to increased TGF-β1 signaling. In another study from the same group, Rojbani et al. compared α-TCP to β-TCP and HAP with and without loading simvastatin.^42^ As previously shown, the simvastatin-loaded α-TCP exhibited comparable bone formation as the simvastatin-loaded β-TCP, but was significantly greater than simvastatin-loaded HAP and each empty particle type. Interestingly, by 8 weeks, less than 10% of the α-TCP microparticles with and without simvastatin remained in the defect site compared to β-TCP (∼35%) and HAP (∼50%). This finding could be primarily driven by the solubility of the α-TCP, and its dissolution could be further enhanced while simultaneously delivering simvastatin in the defect site. More recently, α-TCP was utilized to dual deliver growth factors for large size calvarial defects. In work by Kim et al., microcarriers in two different size scales (small (100–200 μm) and big (300–500 μm)) were used to deliver VEGF and BMP-2, respectively.^43^ Individually, small VEGF-loaded microcarriers and larger BMP-loaded microcarriers both had large burst releases of their respective growth factors, 73% and 51.6%, and sustained releases upward of 3 weeks. When the two microcarriers loaded with their respective growth factors were mixed 1:1, the total release of VEGF and BMP-2 was lowered to 54% and 39%, while maintaining first-order release kinetics. Individually, the small and big microcarrier porosities were 29% and 36%, respectively, but when mixed the final porosity was 14% suggesting that the decrease in release was driven by the porosity of the microcarriers. When implanted in calvarial defects, the mixed microcarrier system significantly improved defect healing size compared to empty defects. However, after the experimental period, microcarrier size and number were not reduced in the defect site. This suggests that the rate of bone formation was greater than that of particle dissolution. Overall, TCPs are an exciting class of CaPPs that can exhibit tuned dissolution with the potential to match bone growth. However, additional work needs to be carried out to optimize the balance between drug release and particle dissolution for optimal tissue repair and/or regeneration.

A less common but highly tunable CaP phase is octacalcium phosphate (OCP).^44,45^ OCP is a hypothesized precursor to biological apatite that possesses a similar structure to HAP but with greater capacity for protein absorption, and improved solubility, contributing to improved loading and drug release.^46^ Forte et al. coprecipitated bisphosphonate drugs into OCP microneedles that exhibited high loading capacities of alendronate and zoledronate.^47^ Although OCP loaded high amounts of each drug, their inherent interaction with the crystal lattice affected crystal size dimensions and the release behavior of each bisphosphonate. For zoledronate coprecipitated OCP microneedles, the X-ray diffraction data showed the presence of another CaP phase. In addition, the release of zoledronate from the particles was drastically lower than alendronate in similar release conditions. The authors speculated that zoledronate formed hydrogen bonds with the OCP structure, whereas alendronate is loosely bound at the surface causing higher release rates from the OCP microneedles. Using a similar form factor of microneedles, Li et al. coprecipitated ibuprofen into OCP particles or HAP.^48^ They found that OCP exhibited a higher loading capacity for ibuprofen than HAP and slower release rates that extended longer than HAP by 10 hours (h). Using the Higuchi model, both CaP phases exhibited two linear release lines suggesting a two-step release behavior. Because of the dynamic conditions of the release, including protonated/neutral ibuprofen species, simple diffusion of the drug, and other desorption–absorption processes, the mechanism driving this two-step release effect could not be identified. OCP have many synthesis methods that can be combined with a variety of drugs including bisphosphonates,^47^ ibuprofen,^48^ antinoeplastics,^49^ and antimicrobials.^50,51^ Additionally, OCP has a great capacity for regenerating bone that is compositionally similar to intact bone when implanted in a calvarial defect model through its improved biodegradation.^52,53^ Thus, using OCP alone or with other phases of CaP for craniofacial bone drug delivery is a potential area to explore for improved healing outcomes.

#### 2.1.3 Biphasic CP (BCP) Microparticles

Biphasic calcium phosphate (BCP) microparticles are a promising strategy for filling craniofacial bone defects due to its improved injectability allowing BCPs to fill complex bone defect geometries compared to cements and other CaP particles of nonuniform geometries.^54^ In addition, BCP has improved dissolution relative to monophasic CaPs owing to the phases involved, with one CaP phase typically exhibiting faster dissolution than the other. The most common BCP combination is less soluble HAP and more soluble TCPs. By controlling the HAP/TCP ratio, researchers can tune particle dissolution and improve the bioactivity and resorption of particles that facilitate the ingrowth of new bone. Other critical attributes that influence dissolution and resorption are low porosity, surface area, high crystallinity, and large particle size. Although these factors play a critical role in osteoconductivity and osteoinductivity, they also influence drug delivery from BCP microparticles. Extensive reviews have examined this topic and readers are referred to them.^55,56^

**Figure 3:**
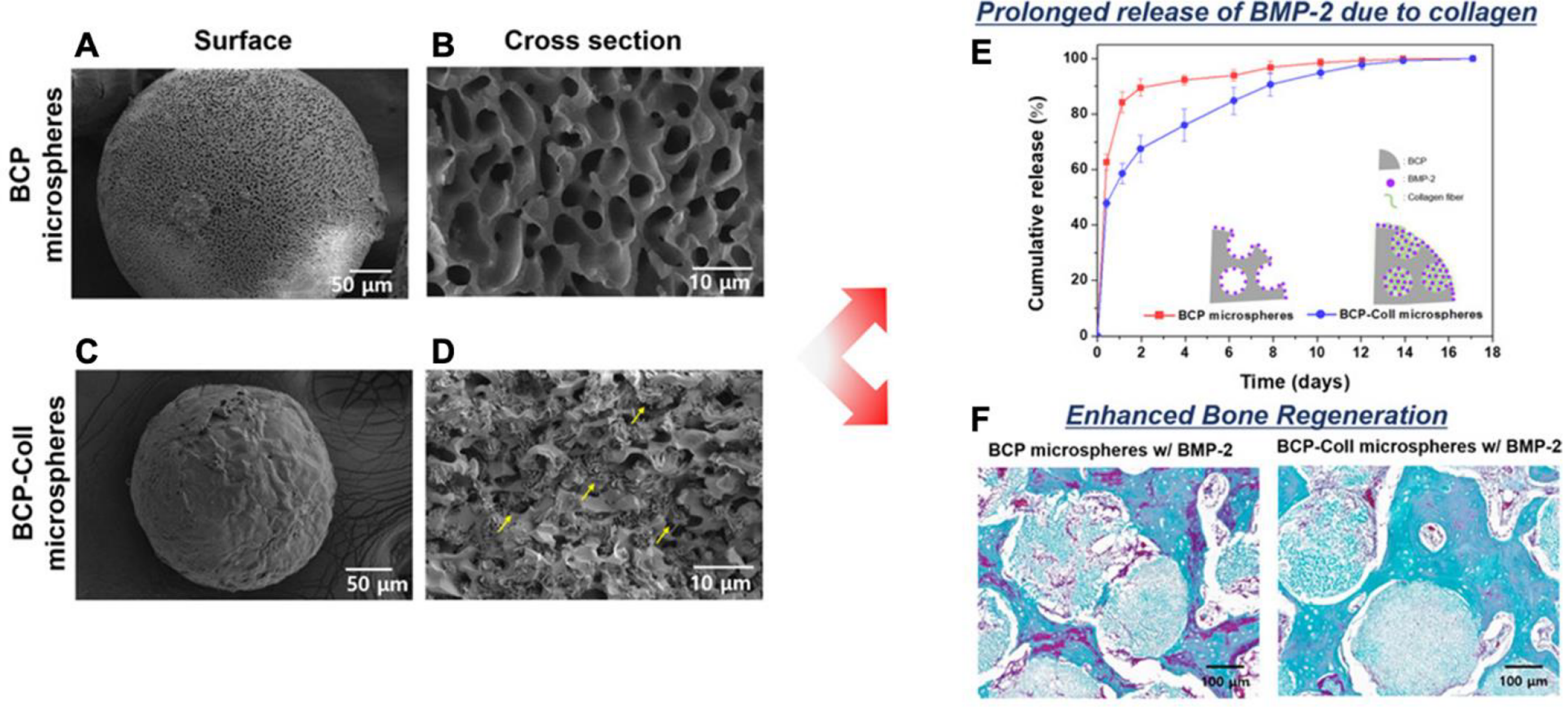
Biphasic microparticles with collagen infiltration for BMP-2 drug delivery. (A–D) SEM of porous BCP microspheres (A, B) and porous BCP microspheres with collagen filtration (BCP-COLL) (C, D). (E) Percent release of BMP-2 from porous BCP microspheres (red) and BCP-COLL microspheres (blue). (F) Goldner trichrome staining of demineralized tissue sections postimplantation. Reprinted with permission from ref (58). Copyright 2022 Elsevier.

Work from Zarkesh et al. examined the effects of BCP microparticle surface topography that influenced porosity to modulate small molecule and protein release.^57^ Using a wet precipitation method of CaP precursors in the presence of varying concentrations of ethylenediaminetetraacetic acid (EDTA), calcium-deficit HAP microparticles with distinct long and short sheets could be formed. With the addition of thermal treatment, long- and short-sheet BCP microparticles were synthesized while preserving crystal morphology and clearing the EDTA template. These distinct topographical features led to differences in porosity and surface area that influenced the release of Bovine Serum Albumin (BSA) but not of small molecule dexamethasone. Although the long-term release was attainable for dexamethasone, there were no alterations in its release kinetics. In contrast, BSA exhibited lower release rates on the long sheet BCP versus the short sheet BCP presumably due to a higher porosity and surface area that exposed more a-planes rich in calcium ions. As an alternative method to tune release from BCP microparticles, Seong et al. developed porous HAP/β-TCP BCP microspheres using camphene as a pore generator^58^ (Figure 3). When loaded with BMP-2, BCP microspheres exhibited a large initial burst release of 85% within the first day. With the infiltration of collagen into the pores of BCP microspheres, the release could be controlled and reduced the burst release to 57% with sustained release for 3 weeks. This effect was primarily driven by collagen’s ability to further adhere BMP-2 in the porous compartment and act as a sink to slow the release. The BCP-collagen spheres further showed an enhancement in bone healing in patellar defect models due to the increased BMP-2 loading and sustained release.

In a different approach, Honda et al. used BCP microspheres to immobilize an antimicrobial peptide, protamine, on BCP surfaces to inhibit bacterial adhesion.^59^ Leveraging the negatively charged BCP and positively charged peptide, the BCP microspheres exhibited high loading efficiency. The protamine-loaded BCP microparticles were able to prevent bacterial adhesion and biofilm formation of both *Staphylococcus aureus* and *Escherichia coli*, with sensitivity seen with *S. aureus.* Thus, BCP exhibits a high affinity for a range of molecules for either extended-release or entrapment to induce local drug effects. Additional work investigating less commonly used CaP phases in BCP microparticles could be of interest based on the needed release kinetics for certain bone regeneration applications.

### 2.2 CaP Nanoparticles for Craniofacial Bone Regeneration

CaPPs as nanoparticle drug delivery systems have garnered extensive interest due to their tunability and facile loading of therapeutic agents such as small molecules,^60^ genes,^61^ proteins, and peptides.^62,63^ Advantages in CaP nanoparticle drug delivery are higher drug adsorption, dissolution rates, surface-to-volume ratios, and control of morphologies for sustained and local delivery.^64,65^ Based on these advantages, studies have focused on using CaP nanoparticle systems primarily in the phases of HAP and TCP. Moreover, further research demonstrates the use of other less commonly used monophasic CaP phases for drug delivery. Here, we will review the recent advances in CaP nanoparticles for drug delivery and their implication in craniofacial bone repair (Table 2).

#### 2.2.1 HAP Nanoparticles (nHAP)

nHAP has strong appeal for drug delivery as it is structurally similar to nanostructured HAP crystals found within the collagen helices of bone. Additionally, nHAP features such as shape, crystal topography, pore size, and polymer surface functionalization can be modulated to tune drug delivery.^66^ As a function of shape, Swain and Sarkar investigated nHAPs of spherical, rod, and fibrous morphologies and reported exceptional loading of BSA for each nanoparticle containing 26.5, 28, and 25.7 mg/g, respectively.^67^ The highest loading observed for nHAP rods was attributed to higher surface area, calcium/phosphate ratios, and its semicrystalline behavior due to the pH and temperature of the synthesis. Consequently, nHAP rods exhibited 75% of BSA release within the first 96 h followed by slow, sustained release up to 240 h. At 96 h, sphere and fibrous nHAP exhibited a modest decrease in BSA release. Using a similar approach, investigators in the Desai lab evaluated how shape and size of CaP nanoparticle conglomerates effected antibiotic drug delivery *in vitro*.^68^ Here, nanoparticle morphologies were obtained by synthesizing various CaP phases: DCPA (flaky and elongated orthogonal particles), OCP (brick-like particles), and HAP (spherical particles). Using fluorescein as a model drug, HAP exhibited the highest loading capacity (1 μg/mg of particle). Interestingly, there were no discernible effects on release kinetics when only small portions of the collection media were sampled. Whereas when the collection media was fully replaced daily, the elongated orthogonal, brick, and spherical particles exhibited near zero-order release kinetics presumably due to the negated dissolution effects either from urea or supersonication during the synthesis. Moreover, the higher surface area of the HAP spherical particle exhibited the best release and subsequent antimicrobial activity when loaded with clindamycin. Here, the contributions of surface area can be appreciated as it strongly facilitates improved loading and release kinetics. Therefore, additional strategies focusing on surface area modifications may prove useful to tune drug delivery.^69,70^

nHAP has been used as a template for surface functionalization with various polymers including PLGA,^71^ PEG,^72^ and lipids.^73−75^ Investigators in the Desai lab showed that PLGA can be reliably coated onto nHAP without inducing coalescence, which could significantly reduce the burst release of clindamycin seen from nHAP alone. Interestingly, only a nominal amount of clindamycin (<0.3 mg/mL) was released until day 5. Afterward, a rapid release and plateau was observed due to the swelling-induced erosion of the polymer and the subsequent release driven by simple diffusion. Using lipid coatings for nHAP controlled delivery, Placente et al. used lipid membrane mimetic coating, LS75, that was optimized to maximize loading onto nHAP surfaces.^75^ Using these coatings, the loading efficiency of two small molecules ciprofloxacin and ibuprofen (36% and 22%) versus noncoated nHAP (11% and 3%) was improved. When they assessed the release behavior from physiological (7.4), neutral (6.2), to acidic (4.2) pH, drastic changes were observed that exhibited the pH-responsive controlled delivery. At physiological and neutral pH, ibuprofen exhibited initial burst releases within 5 h but significantly lowered at acidic pH due to entrapment in the lipid membrane via decreased solubility. Ciprofloxacin, a zwitterionic molecule, exhibited elevated drug release at physiological and acidic pH but lowered release at neutral pH. Thus, this study was able to demonstrate the utility of biomimetic functionalization of nHAP surface with stimuli-responsive release behavior.

Although there have been many studies showing the tunability of nHAP, some hurdles remain for this to be effectively used for craniofacial bone delivery. As seen from Maduhumathi et al., CaP nanoparticles can be applied to large-size calvarial defects without the need for a carrier system, but their retention is diminished in the defect site presumably through the higher dissolution rate attributed to their high surface area.^76^ To overcome this limitation, various studies have created composite materials embedding nHAP in scaffolds or hydrogel matrices to facilitate retention. Nonetheless, nHAP show promise in a wide variety of biomedical applications, especially in the craniofacial complex.

#### 2.2.2 Other CaP Nanoparticles

α- and β-TCP, ACP, DCPA, and calcium-deficient hydroxyapatite (CDHA) are among other CaP nanoparticle drug delivery systems described in recent literature that have shown advantages for craniofacial bone drug delivery (Table 2). The Desai lab has extensively studied ACP and shown that it possesses significant tunability for small molecule drug delivery. Uskokovic et al. showed that ACP was the most effective phase of CaP for loading clindamycin when compared to other nanoparticle phases: HAP, DCPA, and MCPM. This was attributed to ACP’s amorphous surface that allowed for restructuring of the crystal lattice through dissolution/recrystallization behavior.^60^ Also using ACP for small molecule drug delivery, Qi et al. demonstrated that ACP carriers can be synthesized to possess pH-dependent delivery for doxorubicin.^77^ ACP carriers exhibited ∼55% loading efficiency and a dramatic change in release behavior at neutral pH, where less than 5% was released over 190 h but increased to 35% at acidic pH. Furthermore, Sun et al. examined ACP nanoparticles as drug carriers for alendronate and showed high loading efficiency (88%) with sustained release upward of 22 days with only 25% released by the final time point, demonstrating a highly effective ACP nanocarrier for the localized and sustained release of alendronate.^78^

**Figure 4:**
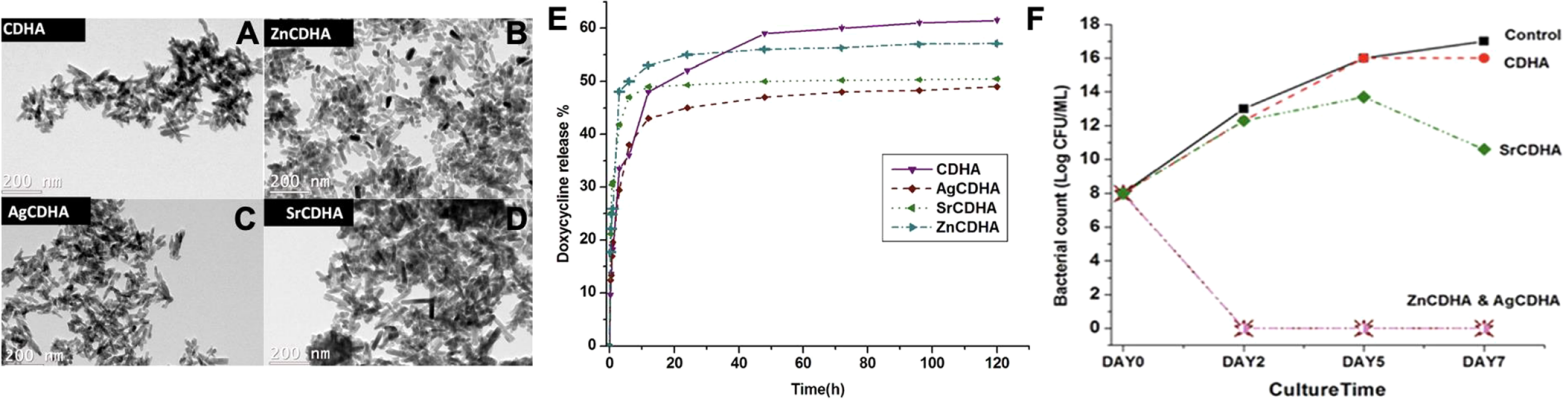
Dual-mode antimicrobial and ion delivery. (A–D) TEM of CDHA (A), zinc (Zn^2+^)-substituted CDHA (B), silver (Ag^1+^)-substituted CDHA (C), and strontium (Sr^2+^)-substituted CDHA (D). (E) Doxycycline release from ion-substituted material. (F) Bacterial counts overtime after incubation with Ion-substituted CDHA. Reprinted with permission from ref (80). Copyright 2022 Frontiers.

CaP nanoparticles’ solubility and porosity have been shown to influence BSA loading and release profiles, especially for α- and β-TCP nanoparticle systems. By examining loading efficiency of BSA in DCPA, β-TCP, and HAP nanocarriers over 24 h, Lau et al. found that BSA loading and release is directly linked to CaP solubility and pore size. DCPA particles exhibited the highest loading of BSA (50 wt %), and β-TCP exhibited 2.7 times greater loading capacity of BSA (41 wt %) over HAP. In addition, the release behavior could also be attributed to the particle solubility where β-TCP exhibited the largest cumulative release.^79^ Moreover, CDHA nanoparticles offer an appealing CaP system for antibiotic drug delivery and antimicrobial activity due to their ability to undergo ion substitutions. Kumar et al. demonstrated that CDHA nanoparticles exhibited doxycycline loading of 68% and a release of 69% over 3 days. These particles exhibited the highest drug loading compared to Ag^1+^, Sr^2+^, and Zn^2+^ ion substituted CDHA nanoparticles, suggesting that an increase in ion substitutions results in a decrease in drug loading (Figure 4). Despite this, ion substituted CDHA were able to deliver adequate levels of doxycycline while decreasing bacterial growth due to the antimicrobial ion substitutions showing the utility for CDHA dual delivery functionality.^80^

#### 2.2.3 BCP Nanoparticles

BCP nanoparticles have been utilized to leverage findings from both TCP and HAP nanocarrier systems. By incorporating biocompatible agents during synthesis, BCP nanostructures (mainly TCP and HAP combinations) exhibit enhanced protein adsorption and tunable release kinetics due to greater control over final nanostructure, pore size, and distribution.^79,81^

Using a strategy to fabricate porous BCP ceramic spheres with nanocrystalline features, Li et al. employed the use of a hybrid alginate-gelatinizing approach with a microwave sintering method to fabricate BCP particles with nanotopographical features of biphasic HA and TCP.^82^ Utilizing a mandible critical-size defect model in rabbits, the BCP nanocrystalline structure improved the osteoinductivity and bone regeneration due to high contents of CDHA phase, abundant micropores, and large surface area which prompted superior cell attachment, spreading, and proliferation.^82^ It is important to note that the authors did not investigate the BCP particles as drug delivery vehicles but have effectively highlighted BCP nanoparticles have promising results for mandible regeneration. Additional studies could be of interest to examine their ability to deliver drugs for similar applications. Thus, while multiple groups have demonstrated the use of BCPs as fillers to bone void defects,^82,83^ others in the field have shown that BCPs hold great regenerative potential not only as osteoinductive and osteoconductive cements and fillers but also as drug delivery vehicles.

Work highlighting the synergistic use of multiple CaP phases as nanocarriers have increased over the years with many describing combinatorial drug delivery carriers with antibacterial, anti-inflammatory, and bone-regenerative dual-drug loaded capabilities. Madhumathi et al. demonstrated a multidrug loaded CDHA/β-TCP and HAP/β-TCP nanocarrier systems for the codelivery of antibiotics and ibuprofen. CDHA exhibited a maximum loading of 70% versus 55% loading in the HAP. Compared to the β-TCP portion, which demonstrated a loading efficiency of 80% for ibuprofen, loading efficiency did not appear to be surface area dependent for either concentration.^76^ The loading and release of ibuprofen from β-TCP nanoparticles exhibited a burst release over a 12 h period which was attributed to the slow-release rate of the adsorbed ibuprofen from the TCP surface over 5 days. The maximum release profiles were shown to be between 24 and 48 h with trends fitting the Higuchi model of diffusion, suggesting that ibuprofen release primarily occurred via diffusion from the β-TCP nanoparticles.^76^

### 2.3 Calcium Phosphate Polymer-Induced Liquid Precursors (PILPs) for Craniofacial Bone Biomineralization

At the nanoscale, 2–4 nm thick HAP platelets infiltrate and embed type-I collagen fibrils providing adequate stiffness and toughness for the bones to withstand different mechanical loads.^84,85^ In diseases that reduce bone mineral density, such as osteoporosis, the lack of HAP increases the risk of fracture repair, reduces bone quality, and increases susceptibility to alveolar bone loss. In addition, loss of bone mineral density increases the risk of periodontal disease which can lead to tooth loss. Although several surgical and bone regeneration methods have been studied, these methods lack the ability to regenerate hypo-mineralized bone by remineralizing the collagen fibrils. Thus, developing methods that deliver the necessary components to remineralize collagen at the nanoscale and reproduce the mechanical properties and composition of native bone could address significant complications seen in oral health.^86^

**Figure 5:**
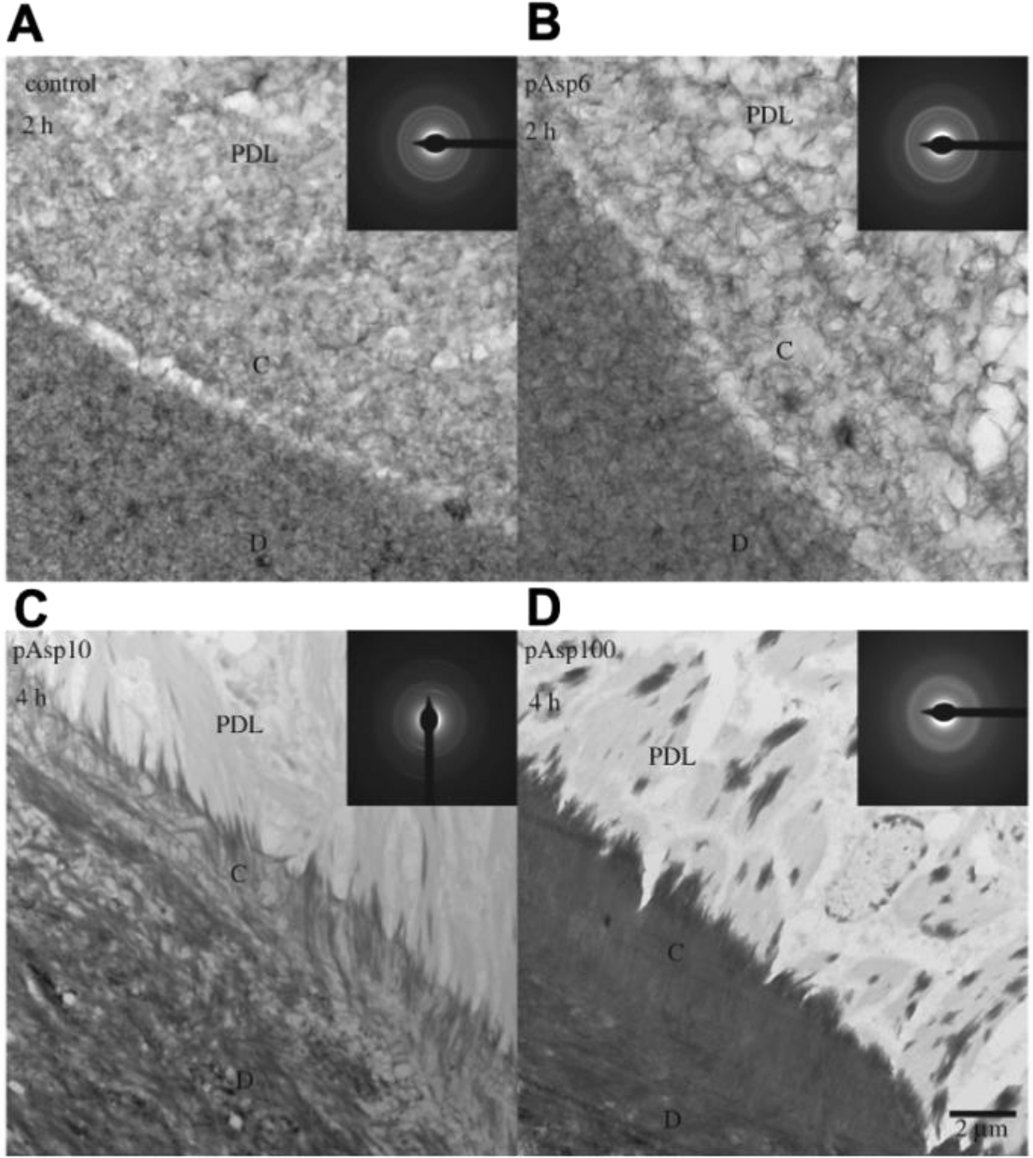
PILP mineralization on periodontal tissues. (A–D) TEM of dentin, cementum, and periodontal ligament tissue sections remineralized with (A) CaP with no pAsp, (B) pAsp (MW 6), (C) pAsp (MW 10), or (D) pAsp (MW 100). Insets are selected area electron diffraction (SAED) of cementum. Used with permission from ref (90). Copyright 2018 The Royal Society, permission conveyed through Copyright Clearance Center, Inc.

An alternative class of ACP nanodroplets has been formed through a bioinspired polymer-induced liquid precursor (PILP) mineralization method (Table 3). In this PILP mineralization method, charged polymers like polyaspartic acid (PASP), poly(acrylic acid) (PAA), and poly(allylamine hydrochloride) (PAH) are used to chelate and stabilize CaP in their liquid-like amorphous state which emulates the process seen by noncollagenous proteins *in vivo*.^87^ After formation, the PILP nanodroplets infiltrate collagen fibrils and form-oriented HAP crystals that exhibit diffraction patterns similar to that of natural bone.^88^ Recently, Yao et al. utilized ACP-PILPs to treat osteoporotic bone in long bones.^89^ Using PAA and PASP as the chelating agents, nanoclusters containing high calcium concentrations were formed that could penetrate and remineralize collagen and significantly improved osteoporotic bone mechanical properties *in vivo*. In a similar approach using PASP, Quan and Sone evaluated how PASP chain length affected remineralization of demineralized periodontal tissues *in vitro*(90) (Figure 5). High molecular weight (MW) PASP can selectively remineralize dentine and cementum while leaving the naturally nonmineralized periodontal ligament unaffected. However, CaP solutions containing no charged polymers and lower MW PASP induced random HAP mineralization on periodontal tissues. This finding was attributed to two factors: 1) association of higher MW PASP chains slowed down ACP formation and subsequent HAP phase transformation and 2) the presence of tissue-associated nucleators of HAP precipitation increase the kinetics for intrafibrillar mineralization in dentin and cementum. Thus, the combination of preventing solution precipitation and kinetically favored intrafibrillar delivery of ACP-PILPs by high MW PASP is favorable for periodontal ligament tissues. This finding may help guide future applications of PILP mineralization for periodontal applications that suffer from demineralization like periodontal disease and orthodontic relapse.^91,92^

**Table 3:** Summary of PILPs for Bone Regeneration and Biomineralization

chelator	kDa	size	defect model	associated carrier
PASP and PAA	9–11 and 450^89^	1 nm^89^	osteoporotic bone^89^	none
PASP	2–11^88^	1 nm^88^	demineralized periodontal ligament^90^	none
	0.708–14^90^	20–30 nm^90^		

PILP mineralization methods showcase a promising approach for treating diseases exacerbated by a lack of mineral by delivering amorphous precursors that can subsequently become crystalline HAP in the intrafibrillar compartment of collagen. Most PILP research is on artificial *in vitro* systems allowing for mechanistic work underpinning how these nanodroplets form, prevent solution mineralization, and direct intrafibrillar mineralization.^93^ Importantly, when attempting to create CaP delivery methods for remineralizing craniofacial bones, aspects of the periodontal ligament enthesis,^94^ noncollagenous proteins involved in mineral inhibition/nucleation,^95^ and cellular contributions will need to be considered. Nonetheless, continued work in this emerging area in relevant animal models will elucidate how PILPs can penetrate deep craniofacial tissues, any inherent toxicity effects of the PILP additives, and whether this approach can recapitulate the dynamic composition and mechanics of craniofacial bones.

## 3 CaP Delivery Systems for Dentin Regeneration and Biomineralization

Dental decay is the leading disease in the field of dentistry affecting ∼2.4 billion worldwide, which accounts for nearly 35% of the entire world’s population.^96^ Typically, superficial cavities affecting the enamel can be treated and arrested with typical fillings. However, deep lesions affecting the dentin and pulp of the tooth have severe side effects including hypersensitivity, inflammation, and/or infection. These insults to the dentin–pulp complex can increase patient pain and, if left untreated, tooth loss and serious damage to surrounding tissues. Currently, there are only two strategies available to treat deep cavitated lesions.^97^ First, vital pulp therapy harnesses the healthy stem cell population within the dental pulp to induce odontoblast-like cells to differentiate, replace the damaged population, and form a dentinal bridge with scar tissue. Second, if the dentin–pulp damage is irreversible, then a root canal is performed that causes the loss of the vasculature, innervation, and stem cell population which drastically reduces the longevity of the tooth. Given the prevalence and negative outcomes that occur with deep cavities, from a tissue engineering perspective, biomaterial formulations carrying relevant cargos to drive remineralization of dentin and/or stem cell differentiation are of the utmost importance.^98^ A variety of materials for delivery systems have been employed including polycaprolactone,^99^ chitosan,^100,101^ PLGA,^102,103^ mesoporous silica,^104^ hyaluronic acid,^105^ collagen,^106^ and CaP-based cements.^107,108^ Given the environment of the tooth and loading demand for mastication, particle systems have fewer advantages here versus cement composites. Thus, we aim to review the recent advances in CaP form factors (i.e., microparticles, nanoparticles, and PILPs) alone or that are embedded in cement and/or scaffolded systems that aim to deliver favorable factors to induce dentin–pulp repair or facilitate the remineralization of demineralized dentin (Table 4 and 5).

**Table 4:** Summary of Calcium Phosphate Micro- and Nanoparticles for Dentin Regeneration and Biomineralization

phase	size	defect model	preparation method	method of release modulation	associated carrier
HAP	100–1 μm	pulp capping^109^	wet precipitation^109,111^	none	multilink automix^111^
		*ex vivo* tooth^111^			
	>200 nm	*ex vivo* dentin^113,115,117^	wet precipitation^115^	particle size^113^	calcium carbonate dentifrice^113^
			polymerization precipitation^117^		
			commercial^113^		
	200–1 nm	*ex vivo* dentin^116^	sol–gel^116^	none	β-TCP microparticles^114^
		*in vivo* human^114^			
ACP	>200 nm	*ex vivo* dentin^123^	wet precipitation^113^	ion substitution^123^	none
	200–1 nm	*ex vivo* dentin^119,120,122^	niosome templating^119^	surface structure^119^	BisGMA-TEGMA^120^
			spray drying^120^	surface area^122^	
				wet precipitation^122^	

**Table 5:** Summary of PILPs for Dentin Regeneration and Biomineralization

chelator	kDa	size	defect model	associated carrier
PASP	27^128,129,132,133,137,140,141^	10–20 nm^140^	artificial dentin lesion^128,129,132,137,140,141^	self-etch adhesive^137^
		20–80 nm^137^	DSPP knockout mouse^133^	resin-modified glass ionomer^140^
				bioglass 45S5^137^
CMC	400^136^	50 nm^136^	deep caries tooth model^136^	CMC scaffold^136^
PAA and PVPA	1.8 and 24^130^	50 nm^130^	adhesive-bonded dentin^130^	none
PAA	1.8^138^	20–50 nm^138^	artificial dentin lesion^138^	self-etch adhesive^138^
CPP	not provided	not assessed	artificial dentin lesion^139^	toothpaste^139^

### 3.1 CaP Microparticles for Dentin Regeneration and Biomineralization

The use of microparticles for dentin drug delivery has gained some attention in recent years due to its ability to facilitate prolonged ion, drug, and growth factor release. However, only a few CaP phases have been utilized as microparticles for dentin applications due to their inability to penetrate the micrometer-sized dentin tubules. Nonetheless, some groups have examined this CaP form factor for drug delivery (Table 4).

#### 3.1.1 HAP Microparticles

In an attempt to examine CaP phases and odontoblast differentiation, Wang et al. used HAP and OCP to induce reparative dentin formation.^109^ Using microparticles on the size scale of <53 μm, when applied to exposed pulp surfaces of the first molar in rats, HAP and OCP were able to induce reparative dentin formation. Interestingly, the OCP group exhibited more regular dentin tubules that were similar to the previous native dentin wall with intact pulp tissue and odontoblast cell morphology. *In vitro* studies suggest this phenotype from OCP was primarily through a direct cell–material interaction that caused a reduction in proliferation and enhanced differentiation of dental pulp stem cells in part through a *Runx2* signaling mechanism. These findings are exciting for CaPPs systems and provide a mechanistic foundation for other HAP- and OCP-based microparticles for dental pulp capping and potential drug delivery.

#### 3.1.2 Other CaP Phase Microparticles

ACPs are the most commonly used CaP phase for dentin applications owing to the high concentration of calcium and phosphate which facilitate dentin remineralization as well as odontoblast mineralization.^110^ Thus, Sears et al. aimed to develop an antimicrobial delivery system via ACP microparticles embedded in commercially available cement containing no ionic components.^111^ ACP microparticles were loaded with antimicrobial silver nanoparticles and embedded in nonbioactive cement and exhibited similar shear bond strength to other commercially available cements. When used in demineralized dentin surfaces *in vitro*, ACP microparticles significantly improved the remineralization width of the dentin surface when compared to the ion containing cement control. This finding was promising as it provides evidence that cements can be combined with bioactive fillers as well as other drug delivery cargos. Despite the release kinetics not being addressed in the study, other groups have shown that silver nanoparticles and other ionic species can be loaded and released from ACP microparticles for as long as 30 days with some tunable capabilities.^112^ These release kinetics of nanoparticles would be ideal for craniofacial bone applications like osteomyletsis;^60^ however, more *in vitro* studies examining tunable release and minimal inhibitory concentrations of bacterial growth would need to be tested to ensure that embedding in such cement form factors would not impede therapeutic efficacy.

Despite the limited work on CaP microparticle form factors for dentin–pulp applications, this particle form factor has shown the ability to induce odontogenesis, remineralization, and dual-load cargo and ions for subsequent delivery.

### 3.2 CaP Nanoparticles for Dentin Regeneration and Biomineralization

CaP nanoparticles have been studied more extensively in dentin applications due to their ability to penetrate the micrometer-sized dentin tubules.^113^ In addition, the high-surface-area-to-volume ratio enhances drug loading and facilitates drug release.

#### 3.2.1 HAP Nanoparticles (nHAP)

In the unique environment of the oral cavity, nHAP has excellent utility for drug delivery owing to reduced solubility and enhanced affinity for mineralized tissue structures.^114^ To examine this on dentin tissues, Yu et al. synthesized mesoporous silica nanoparticles (MSN) coated with nHAP loaded with epigallocatechin-3-gallate (EGCG) (called EGCG@nHAP@MSN) for sustained delivery to reduce biofilm formation and dentin permeability.^115^ Interestingly, EGCG@nHAP@MSN dramatically improved dentin occlusion and dentin permeability when challenged with mechanical abrasion and acid etching of the dentin surfaces. The EGCG release profile exhibited first-order release kinetics with sustained levels for as long as 1-month (∼65% released at day 30). This release profile was deemed adequate as it was sufficient to decrease bacterial accumulation and biofilm formation on dentin surfaces at 1 week and 1 month of coincubation with *Streptococcus mutans*. Other methods of dentin occlusion have been examined, wherein the size of the nHAP particles and particle agglomeration play critical roles in their ability to infiltrate demineralized dentin matrices.^116^ In a different approach to remineralize dentin, Osorio et al. improved the reactivity of PolymP-*n* nanoparticles by doping them with exogenous calcium, zinc, or doxycycline.^117^ Here, calcium- and zinc-based nanoparticles prevented 100% of fluid flow through dentin tubules by their ability to induce CaP precipitation in the microtubules as early as 7 days. Additional work examining these effects long-term (i.e., >6 months) in challenged conditions (i.e., acid etch) is needed as well as cellular interactions, but these results are promising and further show the advantages of calcium and HAP-based particles to induce remineralization of dentin tubules. In addition, there remain concerns about HAP nanoparticle elimination due to its size via phagocytosis or mechanical influences. To address this potential pitfall, Gamal and Iacono performed a clinical trial wherein patient teeth were EDTA-etched causing exposure of dentin tubules to enhance nanoparticle retention with and without a β-TCP microparticle cocarrier.^114^ The patients that had teeth etched with EDTA and implanted with nHAP significantly improved particle retention relative to non-EDTA treated teeth. Interestingly, with the addition of β-TCP microparticles and EDTA, nHAP had improved cohesion on the surface of the tooth. This concept of nanoparticle retention could play significant roles in drug delivery where lower concentrations of particles and drugs could be used to facilitate dentin repair mechanisms. However, there may be concerns as to whether the extent of dentin exposure would induce hypersensitivity or facilitate the formation of a cavity. Nonetheless, chemical etching has shown promise for drug availability for other applications.^118^

#### 3.2.2 Other CP Phase Nanoparticles

**Figure 6:**
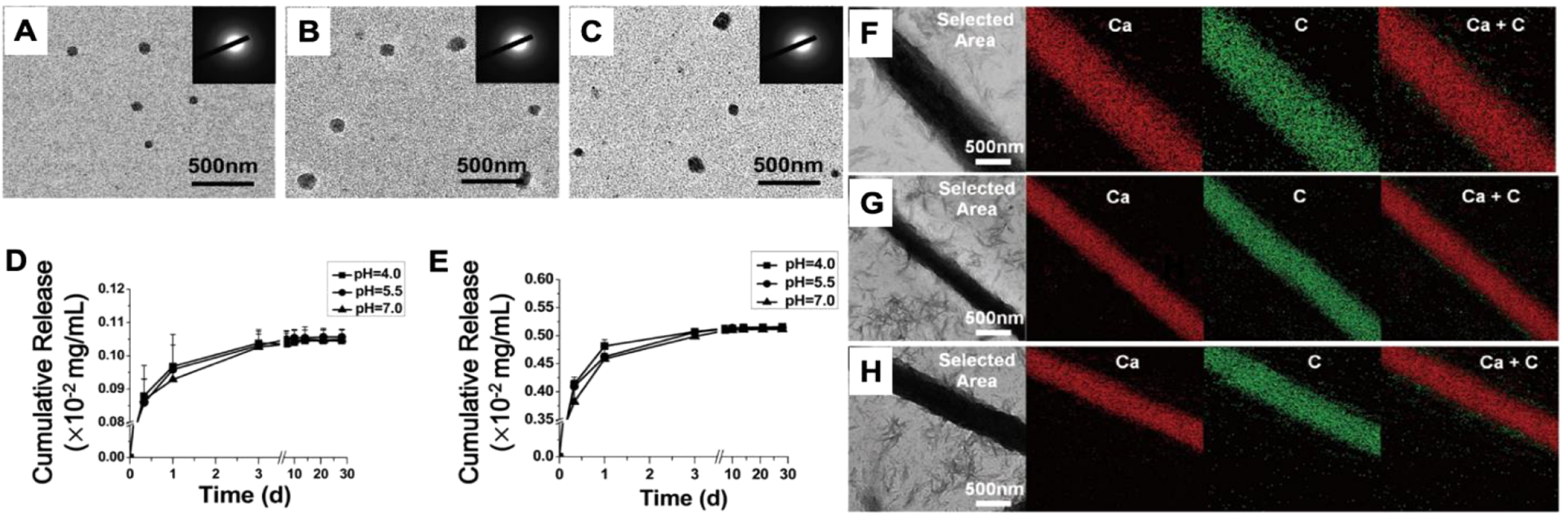
Chlorhexidine-loaded ACP nanoparticles for MMP inhibition and collagen remineralization. (A–C) TEM micrographs of (A) ACP with no chlorhexidine, (B) C-ACP, and (C) G-ACP with SAED indicating ACP. Release of chlorhexidine from (D) C-ACP and (E) G-ACP. Intrafibrillar mineralization as seen with TEM and energy dispersive X-ray spectroscopy maps of collagen fibrils treated with (F) ACP with no chlorhexidine, (G) C-ACP, and (H) G-ACP. Reprinted with permission from ref (119). Copyright 2022 American Chemical Society.

ACP nanoparticles have been used for a combination of drug and ion delivery for dentin applications with various successes in both areas. Cai et al. examined ACP nanoparticles loaded with a matrix metalloproteinase (MMP) inhibitor chlorhexidine, a common antiseptic used in dentistry^119^ (Figure 6). Using chlorhexidine gluconate (G-NCP) and chlorohexidine acetate (C-ACP) during the loading process in ACP formation, the G-NCP exhibited a higher loading efficiency than the chlorhexidine acetate. Although the binding mechanism was not examined in this study to explain the different loading, the release of both chlorhexidine gluconate and acetate exhibited first-order release kinetics for 30 days, with a minor shift due to pH acidification. Examining a single dose of chlorhexidine versus the loaded ACP nanoparticles, the chlorhexidine-loaded ACP nanoparticles exhibited prolonged inhibition of MMP activity and decreased collagen degradation while maintaining their ability to mineralize collagen fibrils. These findings suggest that this chlorhexidine-loaded ACP can facilitate drug delivery, collagen preservation, and remineralization that could be useful in dentin tissue applications. In a different approach using ACP, Melo et al. created a combinatorial dental restoration material containing silver nanoparticles, dimethylaminohexadecyl methacrylate, and ACP to improve the bonding interface of materials and dentin tissue.^120^ The combined effect of the added materials drastically diminished the acidic impact created by biofilm formation which improved the strength of the material–dentin interface. Although a number of materials were added, the authors could not attribute the effect to a single material but suggest that addition of each played a key role in improving the material strength with ACP presumably neutralizing the acid. As previously mentioned, ACP are an excellent source of calcium and phosphate and exhibit high release profiles of these ions relative to crystalline counterparts. Additional doping of ions has been shown to slow down or inhibit the phase transformation of ACP into HAP.^121^ Fluoride is a major cation used in dentistry that causes phase transformations into fluoroapatite that exhibits decreased solubility and is highly resistant to acidic dissolution. Iafisco et al. are the first to report on ACP doped with fluoride ions that can be applied to dentin tubules for occlusion and remineralization of dentin.^122^ In this study, fluoride-doped ACP was stabilized by the presence of citrate ions and by reducing the citrate/calcium ratios, the surface area of the nanoparticles could be increased. The increase in surface area could tune the release kinetics of fluoride and calcium ions in acidic artificial saliva. Fluoride-doped ACP nanoparticles also performed well when used for dentin tubule occlusion; however, the effect was not as pronounced as ACP alone due to fluoride-doped ACP’s quicker phase transition into fluoroapatite. Nonetheless, this study was the first to detail how ACP can be doped with fluoride with tunable release kinetic to potentially tailor the needs for demineralized dentin. Similar studies have found success with other dopants in ACP like magnesium.^123^

Other CaP phase-based cements including α-TCP^124,125^ and BCP^107,126^ have shown promise to deliver drugs and/or embed particle systems to facilitate drug loading and sustained release to improve dentin regeneration. As detailed in this section, ACP is the favored CaP phase due to its high calcium and phosphate release. However, other CaP phases should also be utilized within the field due to their favorable properties for drug release and adequate release of ions for dentin occlusion and dentin regeneration. For instance, OCP would be an ideal approach for dentin drug delivery due to its ability to induce reparative dentin and act as a precursor to HAP transformation.^109,127^ Additional work synergizing drug delivery can lead to significant advances in dentin–pulp complex preservation and repair.

### 3.3 CaP PILPs for Dentin Remineralization

PILP mineralization for dentin has been a pioneering concept since the early 2010s.^128−130^ PILP delivery for dentin applications has been based on concepts in minimally invasive dentistry that describe approaches to facilitate the repair of teeth and restore mechanical properties while preserving healthy components of teeth. With this idea, PILP is a promising approach for restoring the mechanical properties of dentin/teeth, a term known as functional mineralization.^131^ Here, we review literature describing PILP nanodroplet formation for infiltration of collagen in dentin to produce intra- and extra-fibrillar mineralization to create functionalized mineralization in dentin (Table 5).

Burwell et al. used PASP as the chelating agent for ACP stabilization and examined its effects on demineralized artificial carious lesions (∼140 μm depth) on human teeth *ex vivo*.^129^ ACP-PILP treated lesions exhibited an overall 91% improvement in elastic modulus as measured with nanoindentation relative to the nontreated lesion control. Using TEM, collagen fibrils showed a gradual increase in both intra- and extra-fibrillar mineralization over time from 7 to 28 days. However, the spatial mineralization was a concern in the artificial lesion where the inner and outer components were fully remineralized, but only the inner component fully recovered its mechanical properties like native dentin. The authors speculated that one reason this occurred could be due to proteinase activity that was activated during the lesion creation and thus caused irreversible damage to the outer collagen fibril. To test this hypothesis, Nurrohman et al. investigated ACP-PILP delivery in the presence of protease inhibitors for remineralization of artificial dentin lesions on human teeth.^132^ However, when protease inhibitors were added along with ACP-PILP, there was no significant improvement in the mechanical properties of the demineralized dentin relative to nonprotease treated lesions. This finding was attributed to maintained NCP activity that presumably increased dentin tubule occlusion, which did not improve functionally bound minerals to collagen. A similar involvement of NCP and mineral inhibition was proposed and leveraged by Quan and Sone for periodontal ligament ACP-PILP applications.^90^ Despite the complex interplay of factors within the dentin complex, ACP-PILP nanodroplets are clinically relevant and have shown promise in human-related diseases. Using a dentin sialophosphoprotein (DSPP) knockout mouse, a model system that mimics dentinogenesis imperfecta type II (i.e., lack of intrafibrillar mineral) in humans, Nurrohman et al. examined ACP-PILP to remineralize dentin from teeth extracted from DSPP knockout mice.^133^ Interestingly, ACP-PILP were able to restore dentin intrafibrillar mineralization and mechanical properties seen in wildtype mice teeth as assessed with microcomputed X-ray CT and nanoindentation. However, a limitation of this study is the use of ACP-PILP *ex vivo*. Additional studies examining the use of ACP-PILP *in situ* will provide more insight into the translation of this ACP-PILP delivery method and restoration of dentin tissues within this disease context.

**Figure 7:**
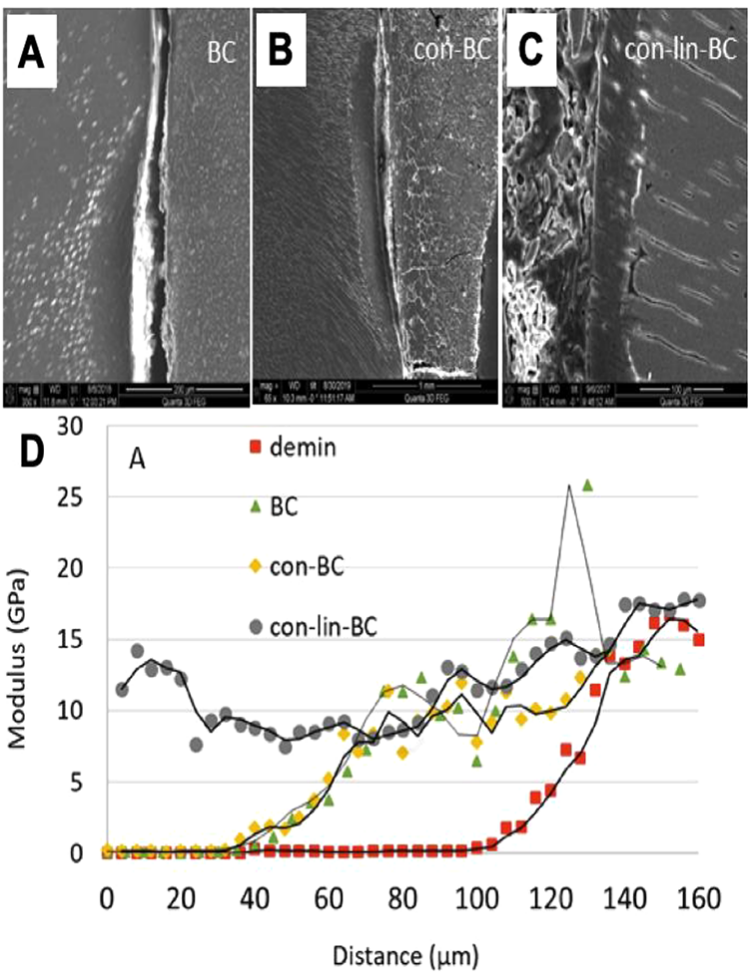
PILP delivery and remineralization of dentin lesions. (A-C) SEM of artificial lesion restored with RMGI control (no PILP) (BC) (A), and PILP conditioner (con-BC) (B), and cement (con-lin-BC)(C). (D) Elastic modulus values versus lesion depth obtained from nanoindentations performed along lines from the lesion surface across the lesion toward normal dentin before (demin) and after treatment. Reprinted with permission from ref (141). Copyright 2022 Elsevier.

To make ACP-PILP more translatable, groups have evaluated ACP-PILP embedded in dental topical formulations, adhesives, or self-setting materials.^134−141^ An interesting approach developed by Shi et al. was based on a mineralized adhesive containing PASP-stabilized PILPs.^134^ Here, they showed that ACP-PILPs could be immersed in the etching material with minimal agglomeration and when applied to etched dentin surfaces, they significantly reduced dentin permeability. This effect was driven by deep penetration of the adhesive into the tubules and the formation of a thick adhesive layer on the dentin surface that was resistant to abrasion and acid attack. The extent to which intrafibrillar mineralization occurred was not specifically examined in this study, but the mineral penetration into dentin tubules and odontoblast processes suggest that some collagen intra- and extra-fibrillar mineralization was achieved. As part of a commercially self-setting material, Babaie et al. developed a novel 45S5 bioglass containing PASP that was able to provide sustained release of PASP and significantly improve both artificial and natural lesions of human teeth^141^ (Figure 7). In this study, a simple mixture of bioglass and PASP (27 and 23 kDa) was mixed in water followed by placing it on the demineralized dentin surface to set and then capped with restoration material. In SBF, the release of PASP from bioglass exhibited low release kinetics, where of the 20 mg loaded, only 120 μg was eluted out by day 30. Although the release was low, this formulation significantly improved dentin remineralization in artificial dentin lesions. However, the effect was not as promising as previously seen with ACP-PILPs alone, suggesting that improved release kinetics of pAsp from bioglass could be more beneficial. Moreover, when natural lesions were treated, remineralization was improved to a lesser extent than artificially made lesions presumably because of the irregular demineralized surfaces, preattained dentin occlusion, and lesion depth.

Although a vast array of work has shown promise in using pAsp-stabilized ACP-PILPs, there are some limitations that still need to be addressed in future works. In all the studies using PILPs for dentin remineralization, the source of calcium and phosphate to drive intrafibrillar mineralization are obtained from the SBF solution that these substrates are immersed. In a clinical setting, submerging teeth in such solutions would be impractical due to the amounts needed and techniques to isolate a singular tooth for such purposes. Achieving more complex composite materials that can either chelate higher concentrations of calcium and phosphate as described by Yao et al.^89^ or embedding additional high ion releasing CaP-based materials could further improve the ACP-PILP formation and subsequent delivery to collagen fibrils.^142^ Overall, these ACP-PILP formulations have shown to be useful for collagen infiltration and remineralization of dentin and could be the future of dental caries therapeutics in dentistry.

## 4 Perspectives and Conclusions

The inherent osteoconductive nature of CaPPs-based materials have been extensively studied within scaffold, cement, and other systems, and their applications assessed for improvements in biocompatibility, biodegradability, and bioresorbability in the craniofacial complex. Optimizing CaPPs in terms of phase composition, size, and shape has been and remains a fertile field for researchers, evolving from simple release systems to more complex CaPP designs that allow for drug delivery to complex craniofacial tissues. Despite these recent advances in the past decade, there remain limitations as well as exciting growth for the field as outlined below.

(1) CaPPs *in vivo* dissolution is still a major problem and has improved in some cases based on enhancing the dissolution profile or enhancing cellular function like osteoclastic resorption through calcium-to-phosphate ratios within the CaP-materials.^143^ Creating more sophisticated BCP blends outside the traditional HAP/TCPs combinations could be more beneficial when targeting sites within the craniofacial complex that heal much more rapidly. In addition, further understanding of the *in vivo* CaPPs degradation profile and the cargo’s biochemical effects on the CaPPs could be an area of interest to synergize drug delivery and host factors for tissue ingrowth. Within this system of delivery, appropriate drug cargoes that modulate the complex interplay of osteoblastic and clastic cells could also be leveraged to induce favorable responses for CaPPs resorption and bone or dentin ingrowth.

(2) While this review focused on CaPPs for craniofacial bone and dentin, the cited literature suggests that these systems can be applied to long bones as well. Although this may be true in some cases, recent studies have shown that this should be investigated more deeply.^144^ Given that craniofacial bones and the dentin of teeth are derived from neural-crest mesenchymal cells while longs bones originate from mesoderm mesenchymal cells, and despite their exhibiting similar mineralization capabilities, the two differ in *in vivo* healing properties when impregnated on CaP-based scaffolds.^144^ This important distinction between cell source and anatomical location exemplifies further appreciation for CaPPs for specific mineralized tissue engineering applications, especially pertaining to the craniofacial complex. By studying the two sources of cells in parallel with similar CaPPs materials, we can start to further understand how we can engineer precision materials with regard to their phase, shape, size, and characteristics to improve treatment outcomes.

(3) With the increased interest in multimodal scaffolds and drug delivery platforms, the new and innovative CaPP synthesis methods reviewed here have enabled researchers to embed such CaPPs with their cargos in larger scaffolds and cements that have proven to be beneficial for mineralized tissue engineering in the craniofacial complex, especially the dentin of teeth. With this approach, the incorporation of CaPPs can further boost current standards in the field that lack a targeted biomineralization component by providing the needed ions. As a whole, composite systems as such can encompass many improved regeneration properties by delivering relevant amounts of morphogenic factors and ions through the CaPP and facilitate enhanced mechanical properties through the carrier scaffold. Additional research in this area of scaffold/CaPP composites with an emphasis on CaPPs synthesis and drug release could lead to more precise therapies targeting specific cells or structures within craniofacial bones and load-demanded dentin of teeth. These concepts could be critical for complex interphases such as the alveolar bone and unmineralized periodontal ligament or the dentin and enamel junction.

(4) For drug and ion delivery to mineralized craniofacial tissues, CaPPs have shown immense potential to fully recapitulate the health of the diseased tissue with great tunability of their release. By harnessing the inherent mineralization mechanisms through different synthesis methods, researchers can leverage features such as size, shape, porosity, and geometries to further optimize release kinetics of drugs and ions for a variety of clinical cases. However, additional work examining the toxicity and long-term tissue effects is important. Recent studies are starting to tease out the effects shape, size, and crystal surface structures have on tissue responses.^145−147^ With the addition of drugs within CaPPs, a more systematic understanding of these tissue responses is critical as the field starts to move toward clinical translation.

Overall, current approaches to CaPPs drug delivery for mineralized tissues of the craniofacial complex are making significant advances as delineated herein. Despite some of the complex intrinsic mechanisms governing the control and release of various drugs, numerous strategies have come into play that have been shown to be primary, if not the sole, driver of the CaPPs delivery methods. Long-term, we continue to expect advances in synthesis methods and mechanisms of drug loading and release that can be applied effectively to the craniofacial complex.

## Abbreviations

HAP: Hydroxyapatite
TCP: Tricalcium phosphate
ACP: Amorphous calcium phosphate
CDHA: Calcium-deficient hydroxyapatite
OCP: Octacalcium phosphate
PILPs: Polymer-induced liquid precursors
PASP: Poly aspartic acid
PAH: Poly acrylic acid
CMC: Carboxymethyl chitosan
CPP: Casein phosphopeptide
PVPA: Polyvinylphosphonic acid
SBF: Simulated body buffer
